# The alveolar edema equation

**DOI:** 10.3389/fphys.2026.1843219

**Published:** 2026-06-19

**Authors:** John C. Grotberg, Francesco Romanò, James B. Grotberg

**Affiliations:** 1 Division of Pulmonary and Critical Care Medicine, Department of Medicine, Indiana University School of Medicine, Indianapolis, IN, United States; 2 Univ. Lille, CNRS, ONERA, Arts et Métiers Institute of Technology, Centrale Lille, UMR 9014 LMFL - Laboratoire de Mécanique des Fluides de Lille - Kampé de Fériet, Lille, France; 3 Department of Biomedical Engineering, University of Michigan, Ann Arbor, MI, United States

**Keywords:** acute respiratory distress (ARDS), alveolar interstitium, alveolar surface tension, cardiogenic pulmonary edema (CPE), positive end-expiratory pressure (PEEP), pulmonary edema clearance, Starling equation

## Abstract

Pulmonary edema is excessive liquid accumulation in the alveolar air spaces and interstitium. This reduces gas exchange leading to increased morbidity and mortality. Clearance of pulmonary edema fluid relies on reabsorption into the alveolar interstitium and then either to the alveolar capillaries or to the lung lymphatics. How it reaches the lymphatics has been a puzzle since 1896. Our 2D model of an interstitial strip resolves that puzzle. We also show that alveolar interstitial pressures differ from values used in traditional medical textbooks. Our approach employs detailed fluid mechanical analysis of the alveolar capillary blood flow, interstitial fluid flow, and the alveolar liquid layer with gas interface and surface tension effects. The governing equations are coupled by Starling equations at both the capillary and alveolar membranes. Traditional approaches only consider the capillary Starling equation. Approximately 80% of the strip is 1D flow directly across the interstitium for either edema or clearance. There, we derive simple equations, usable by physiologists and clinicians, which predict the interstitial fluid pressure and the cross-flow rates (edema or alveolar-capillary clearance). Contrary to conventional tenets, the cross-flow rate magnitude is controlled by the alveolar membrane filtration coefficient, not the capillary membrane. Finally, we derive the critical capillary blood pressure leading to pulmonary edema, i.e., the alveolar edema equation. The latter matches well with clinical data on edema from high blood pressure or ARDS. It can be used to personalize PEEP therapy to prevent edema prophylactically or clear it.

## Introduction

Pulmonary edema is broadly defined as an abnormal accumulation of fluid in lung interstitium and alveoli and may be categorized as cardiogenic or non-cardiogenic in origin. The former is classically seen in congestive heart failure, where elevated lung capillary blood pressure drives fluid across the air–blood barrier. Approximately 1 million people in the USA suffer from this condition annually (Go et al., 2013). Acute cardiogenic pulmonary edema (CPE) has an in-hospital mortality rate of 30%-40% (Amado-Rodríguez et al., 2022). The latter involves damage to that barrier leading, in severe cases, to acute respiratory distress syndrome (ARDS) (Ware and Matthay, 2000, Ware and Matthay, 2005). Prior to the COVID-19 pandemic, approximately 190,000 patients were diagnosed with ARDS annually in the USA (Rubenfeld et al., 2005) at ~40% mortality (Villar et al., 2016). Those numbers skyrocketed in the USA and around the world with the pandemic involving particular features related to the infection (Gibson et al., 2020). They include the acute onset of bilateral alveolar opacities, reduced lung compliance with high shunt fraction, and the classic histopathology of diffuse alveolar damage (DAD) and pulmonary vascular endothelialitis (Ackermann et al., 2020) (Schaller et al., 2020).

Although clinical presentations of these two etiologies share several features (i.e., dyspnea, tachypnea, arterial hypoxemia, and interstitial infiltrates), elements of the patient’s history, laboratory testing, radiographic and ultrasound imaging, and invasive or non-invasive hemodynamics can help differentiate between them. Patients with cardiogenic pulmonary edema and a previous diagnosis of cardiac failure may present with weight gain, peripheral edema, and decreased response to diuretics, whereas those with acute decompensated heart failure, for example due to myocardial infarction, or those with hypertensive crisis, may present with flash pulmonary edema without other signs of volume overload.

Patients with acute lung injury (ALI) and ARDS often present with a clinical history of a known cause of non-cardiogenic pulmonary edema (i.e., pneumonia, aspiration, sepsis, pancreatitis, and others). Given that the initial insult leading to ARDS is often inflammatory in nature, non-specific laboratory serum markers of inflammation such as c-reactive protein, erythrocyte sedimentation rate, white blood cell count, ferritin, and d-dimer may be elevated. More specific markers including interleukin-6 (IL-6), interleukin-8 (IL-8), tumor necrosis factor (TNF), soluble receptor for advanced glycation end products (sRAGE), and angiopoietin 1 and 2 (ANG1 and ANG2) may also be elevated, although these are not routinely measured in clinical practice (Spadaro et al., 2019). Radiographic imaging can also aid in differentiation. Cardiogenic pulmonary edema may be accompanied by an enlarged heart, pleural effusions, smooth septal line thickening, a more central distribution of edema, peribronchial cuffing, and a lack of air bronchograms. In non-cardiogenic edema, the edema is often more patchy or peripheral, air bronchograms are commonly present, whereas pleural effusions, peribronchial cuffing, and septal line thickening are commonly absent. In the past, pulmonary arterial catheters (PACs) were used frequently; however, their use has fallen out of favor. A pulmonary capillary wedge pressure (PCWP), a surrogate for left atrial pressure, ≤18 mmHg supports the diagnosis of non-cardiogenic pulmonary edema, although it is no longer a part of the diagnostic criteria for ARDS. Point-of-care echocardiography and lung ultrasonography have largely replaced the need for PACs. A reduced ejection fraction, E/E′ > 15, or distended inferior vena cava may support elevated intracardiac pressures and cardiogenic pulmonary edema. Currently, lung ultrasonography can be used to diagnose pulmonary edema by the presence of B lines; however, it cannot differentiate the etiology (Ware and Matthay, 2005).

While the focus on pulmonary edema has grown, the field has long lacked a robust mechanistic model, particularly as it pertains to alveolar interstitial fluid dynamics. Such models can be used to structure physiological studies, interpret data, sort diagnoses, promote personalization of interventions, and monitor therapeutic responses. With the experience of a worldwide COVID-19 pandemic costing nearly 7 million lives, there is compelling motivation to establish and investigate a detailed fluid mechanical model of pulmonary edema based on fundamental physics. We recently published a novel model (Grotberg and Romanò, 2023; Grotberg et al., 2025) and here explore its clinical implications, corrections to long held fundamental concepts, and resolution of a century-old puzzle.

## Model results for 2D flow

The history of modeling pulmonary edema includes compartmental approaches of pulmonary interstitial and lymphatic flows utilizing resistance and compliance components like electrical circuit analogues (Goldberg, 1980; Unruh et al., 1984; Drake et al., 1987). These include effects of ventilatory motion (Pearse et al., 2005) with heuristic explanations.

**Figure 1:**
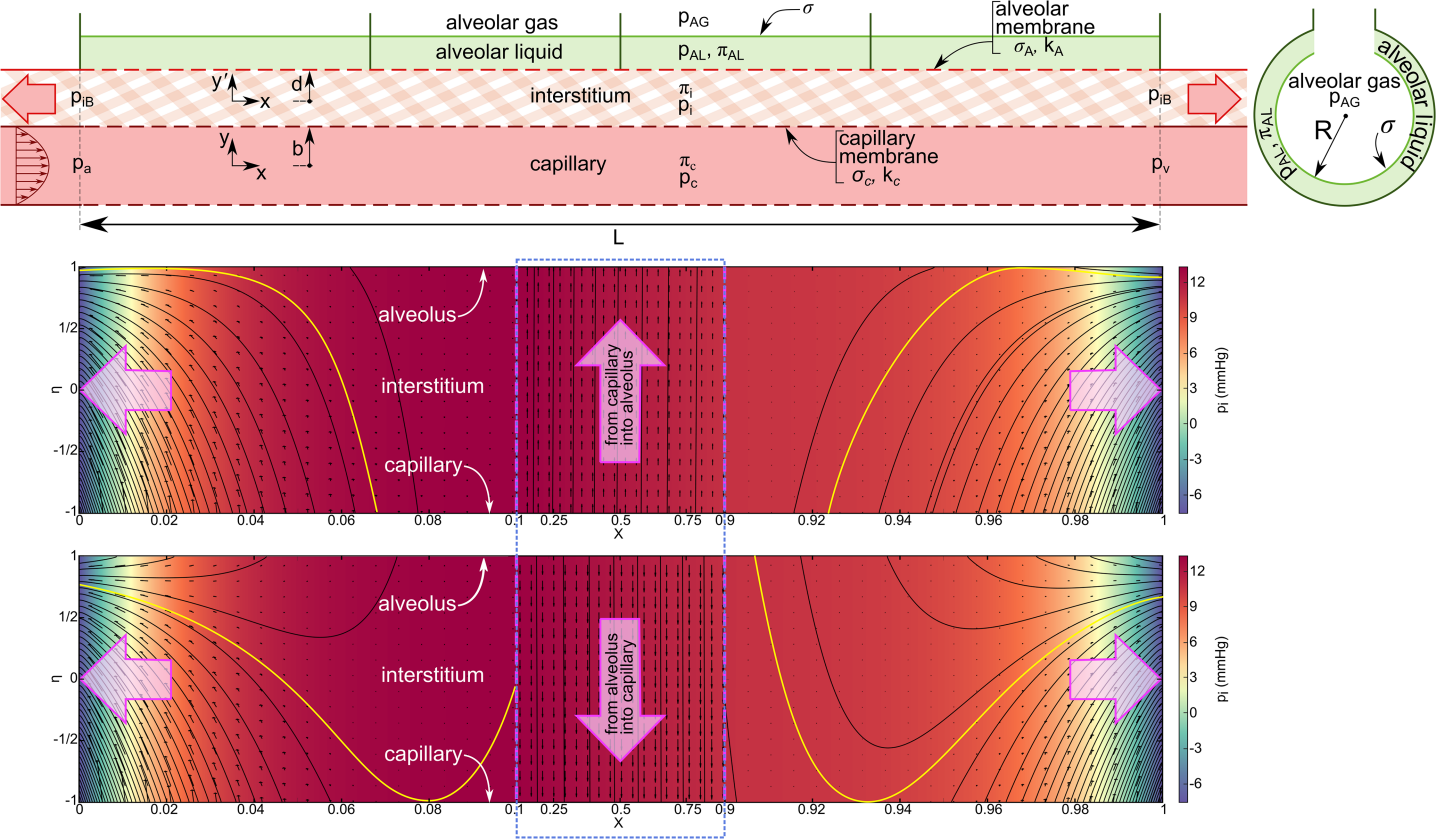
(Top) Our 2D flow model with capillary, interstitium, and alveolar compartments. The interstitial strip length is L=500 μm, and the dimensionless range is 0≤X ≤ 1 where X=x/L. The height is 2d=0.8 μm. (Middle) CPE showing flow from the capillary into the alveolus, i.e., edema. For better visualization, we compress the central region, 0.1≤X ≤ 0.9 (blue box), while expanding the end regions 0≤X ≤ 0.1 and 0.9≤X ≤ 1. In the central region, the streamlines are directly across the layer, whereas for the end regions, the curvilinear streamlines exit at X=0 and X=1. (bottom) CPE with p_AG_ increased from 0 to 15 cmH_2_O (PEEP) which reverses the edema flow to clearance. See text for details.

Our basic flow system is a 2D alveolar interstitial strip separating the capillary from the alveolar compartment [see **Figure 1** (top)]. Blood flow is driven by p_a_>p_v_. It is p_v_ that tracks with PCWP. During edema, fluid (serum) crosses the capillary membrane into the interstitium according to its Starling equation with hydraulic conductivity, k_c_, and reflection coefficient σ_c_. See Equation 1a where the filtration coefficient k_fc_=k_c_A and A is the membrane surface area. Fluid crosses the interstitium and then the alveolar epithelial membrane into the alveolar liquid according to its Starling equation with hydraulic conductivity, k_A_, reflection coefficient, σ_A_, and filtration coefficient k_fA_=k_A_A, see Equation 1b. Clearance involves the reverse flow from alveolus to capillary. There are also flows out the ends going to interstitial regions available to lymphatics. The hydraulic pressures are capillary, p_c_, interstitium, p_i_, alveolar liquid, p_AL_, and alveolar gas, p_AG_. p_AL_ is determined by the surface tension, σ, and alveolar radius, R, through the Law of Laplace $p_{AL}=p_{AG}-2\sigma /R$; see the spherical alveolus insert. The corresponding osmotic pressures are π_c_, π_i_, and π_AL_ as shown.

Alveolar capillary lengths vary from 250 to 850 μm (Townsley, 2012) passing by several alveoli, so our choice is an intermediate value. Based on anatomic–physiologic evidence, the lymphatics are only occasionally in interalveolar septa. Instead, they are primarily in interlobular septa and subpleural regions, up to hundreds of μm from the alveoli (Ware and Matthay, 2005; Weber et al., 2018). That motivates using the subpleural measurements of interstitial fluid pressure as the strip end-pressures, p_i_=p_iB_=−7.35mmHg at X = 0,1 (Miserocchi et al., 1993).

The governing fluid mechanics equations are capillary viscous flow, interstitial porous media flow, and alveolar statics with coupling at the membranes. Solutions yield the displayed streamlines, velocity vectors, and interstitial pressure field, p_i_, which is color coded (Grotberg and Romanò, 2023; Grotberg et al., 2025). **Figure 1** (middle) is CPE with elevated blood pressures p_a_=25mmHg and p_v_=22mmHg. The central region flow, from capillary to alveolus, is edema with maximum p_i_=+12mmHg. The end-region p_i_ gradients in the X-direction drive lateral outflow to interstitial regions available to lymphatics. Exiting streamlines originate primarily from the capillary. These p_c_-driven p_i_ gradients resolve a century-old mystery of alveolar fluid getting to distant lymphatics (Miller, 1896; Staub, 1974; Weber et al., 2018). **Figure 1**(bottom) is the same CPE now with p_AG_ increased from 0 (normal) to 15cmH_2_O (PEEP). This reverses the central region flow to clearance and out the ends where streamlines from the alveolus have an increased share.

**Figure 2:**
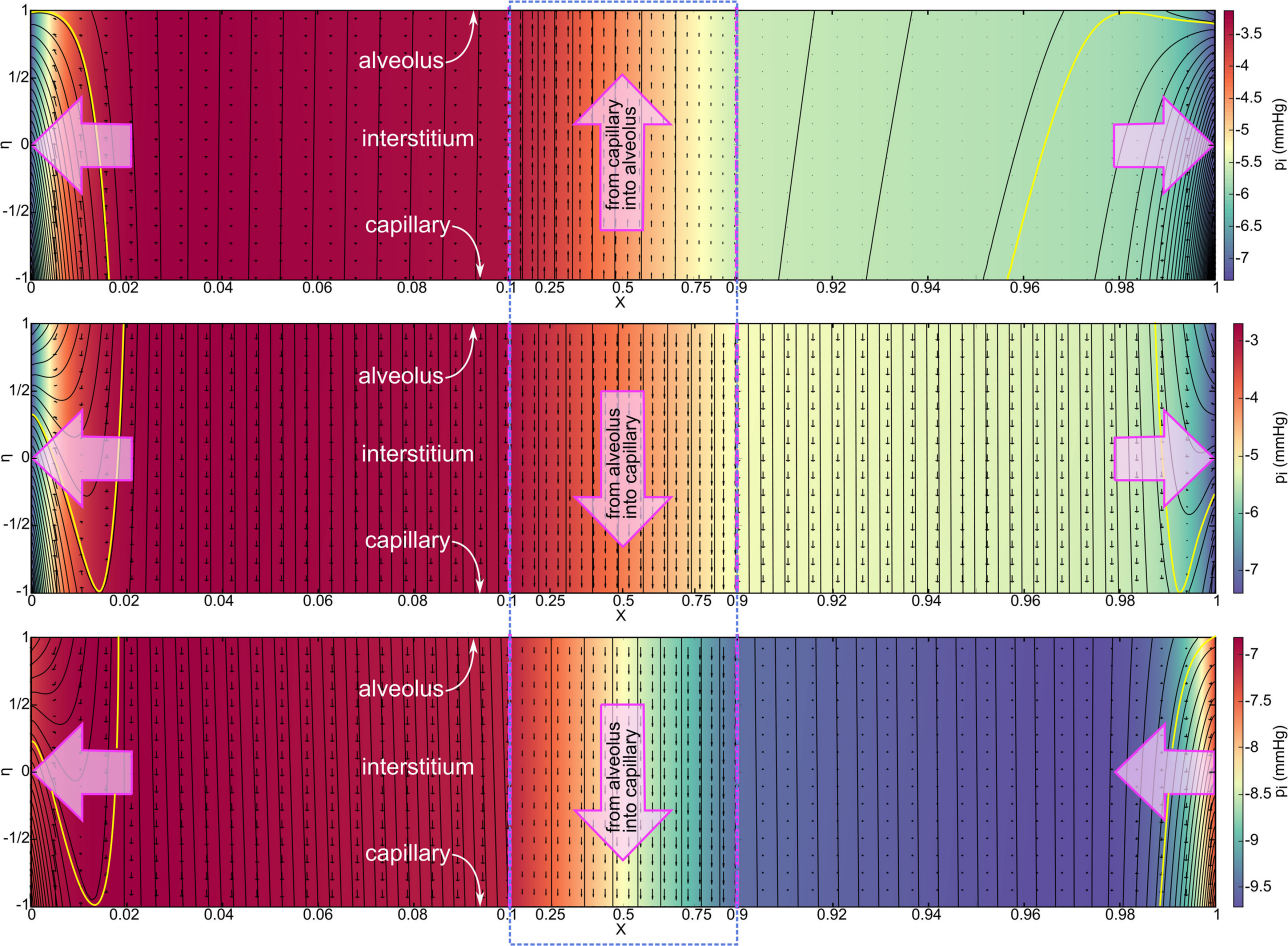
(Upper) ARDS showing edema flow. (Middle) ARDS with increased p_AG_ from 0 to15cmH2O (PEEP) showing clearance. (bottom) ARDS with increased π_c_ from 25 to 30mmHg, also showing clearance. See text for details.

**Figure 2** (Top) represents ARDS with normal blood pressures, p_a_=9mmHg and p_v_=6mmHg, but increased: σ from 4 (normal) to 40 dynes/cm due to interference with surfactant function; π_AL_ from 0 (normal) to 10mmHg due to accumulation of inflammatory products; and k_A_ and k_c_ by factors of 10 due to membrane damage. These ARDS parameter changes align with the biomarkers discussed above. The p_i_ field has maximum p_i_ = −3.5mmHg, much lower than in **Figure 1**, because p_c_ is normal in ARDS but elevated in CPE. This exhibits that capillary p_c_ transmits to interstitial p_i_. Consequently, proposed flows from subpleural to hilar interstitium (Bhattacharya et al., 1984) would have to navigate around this interalveolar septal pressure barrier. **Figure 2** (middle) is the same ARDS but p_AG_ increased from 0 to 15cmH_2_O, reversing the central region flow to clearance. **Figure 2** (bottom) is also ARDS, but with π_c_ increased from 25mmHg (normal) to 30mmHg from osmotic therapy. The central region flow reverses to clearance and the downstream end boundary at X = 1 draws fluid into the interstitial strip.

## Model results for 1D flow

The central region which occupies approximately 80% of the alveolar strip is essentially 1D flow with three resistances in series: capillary membrane, interstitial matrix, and alveolar membrane.

(1) $$\begin{array}{lll} \left(a\right) & J_{Vc}=k_{fc}\left(p_{c}-p_{i}-\sigma_{c}\left(\pi_{c}-\pi_{i}\right)\right) & \text{capillary membrane}\\ \left(b\right) & J_{VA}=k_{fA}\left(p_{i}-p_{AL}-\sigma_{A}\left(\pi_{i}-\pi_{AL}\right)\right) & \text{alveolar membrane} \end{array}$$

The capillary membrane Starling equation is Equation 1a where J_Vc_ is the crossflow rate. Traditional lung physiology describes the fluid balance using Equation 1a. A textbook (Hall and Guyton, 2011) example uses p_i_=−8mmHg, p_c_=7mmHg, π_c_=28mmHg, and π_i_=14mmHg. Assuming σ_c_=1, the result is J_Vc_=k_fc_(+1mmHg) which potentially is edema flow. Preventing fluid entry into the alveolus, the explanation is that an intervening lymphatic vessel suctions the excess fluid. However, from **Figures 1** and **2**, we see that the lymphatic pathway out the ends receives fluid from both capillary and alveolus sources and has no effect in the majority central region.

In terms of clearance flows, the central region has alveolar–capillary clearance, with streamlines directly across the interstitium, whereas the end regions have alveolar–lymphatic clearance. Total clearance is the sum of both. The model alveolar–lymphatic clearance calculates to 10% of the total, so 90% flows directly across to the capillary in the central region. As model validation, this compares well with sheep experiments finding 8.8 to 14.6% lymphatic contribution to total clearance, the rest being alveolar–capillary clearance (Mackersie et al., 1987). In other tissues, lymphatics are plentiful and juxtaposed to capillaries, close enough for suctioning excess filtered fluid. That experience may have motivated the decades-long search for alveolar wall lymphatics serving a similar role. However, the alveoli are quite different since they offer a very large alveolar–capillary alternative route.

Often missing from previous models of pulmonary edema is the Starling equation for the alveolar epithelial membrane, Equation 1b, where J_VA_ is the crossflow rate. Using the conversion factors of 1mmHg=1.36cmH_2_O=1,333dynes/cm^2^, the Law of Laplace becomes $p_{AL}=0.735{\text{ p}}_{AG}-0.0015\sigma /R$ allowing usual physiological units: p_AL_ (mmHg), p_AG_ (cmH_2_O), σ (dynes/cm), and R (cm). Clearly, fluid must cross both barriers for either edema or clearance in the central region. For these direct flows across the interstitium, the interstitial matrix resistance is negligible by comparison with the membrane resistances (Grotberg and Romanò, 2023; Grotberg et al., 2025). Consequently, J_Vc_=J_VA_ in Equation 1 and that lets us solve for p_i_.

(2) $$p_{i}=\frac{\left[p_{c}-\sigma_{c}\left(\pi_{c}-\pi_{i}\right)\right]+\left(k_{fA}/k_{fc}\right)\left[\left(0.735\;p_{AG}-0.0015\sigma /R\;\right)+\sigma_{A}\left(\pi_{i}-\pi_{AL}\right)\right]}{\left(1+\left(k_{fA}/k_{fc}\right)\right)}$$

In Equation 2, we see directly the effects of the capillary, first square bracket, and the alveolus, second square bracket on p_i_. Specifically, increasing either p_c_ or p_AG_ will increase p_i_. The coefficient of the alveolar contribution is multiplied by the ratio k_fA_/k_fc_<<1, since the alveolar membrane offers far more flow resistance than the capillary membrane. Therefore, the capillary is more influential than the alveolus on p_i_ in general. In ARDS both k_fA_ and k_fc_ will increase, potentially leaving this ratio of similar size (Grotberg and Romanò, 2023; Grotberg et al., 2025). For example, normal parameter values from extensive literature review (Grotberg and Romanò, 2023; Grotberg et al., 2025) are p_AG_=0mmHg, σ=4dynes/cm, π_c_=25mmHg, π_i_=10.15mmHg, π_AL_=0mmHg, σ_c_=σ_A_ =0.8, R = 0.01cm, and $k_{fA}/k_{fc}=k_{A}/k_{c}=5x\;10^{-8}cm/\left(mmHg\cdot s\right)/1\;x\;10^{-6}cm/\left(mmHg\cdot s\right)=0.05$. Using the midpoint capillary pressure value p_c_=(p_a_+p_v_)/2 = 7.5mmHg and inserting into Equation 2 yields p_i_=-3.8mmHg. Increasing p_AG_ from 0 to 15cmH_2_O increases p_i_ slightly to −3.3mmHg. Compare that with increasing p_c_ from 7.5 to 23.5mmHg in CPE, which yields p_i_=+11.4mmHg. Equation 2 predictions match well with the color coded p_i_ fields in **Figures 1** and **2** for the central region.

p_i_ is difficult to measure and often restricted to accessible subpleural regions which can be mechanically different from deeper interstitial environments. The ability to calculate p_i_ can open many directions of pulmonary investigations. It also allows us to calculate the flow rate for either edema or clearance. Inserting Equation 2 into Equation 1a we arrive at a predictive expression for J_Vc_ or, equivalently, J_VA_.

(3) $$J_{Vc}=J_{VA}=\frac{k_{fA}}{\left(1+k_{fA}/k_{fc}\right)}\left\{p_{c}-\left(0.735p_{AG}-0.0015\sigma /R\right)-\sigma_{c}\left(\pi_{c}-\pi_{i}\right)-\sigma_{A}\left(\pi_{i}-\pi_{AL}\right)\right\}$$

Because k_fA_/k_fc_<<1, a remarkable feature of Equation 3 is that the flow magnitude across the capillary, J_Vc_, is actually controlled by the alveolar filtration coefficient k_fA_ which multiplies the entire curly bracketed term. That is opposite to traditional thought which focuses only on Equation 1a where J_Vc_ is controlled by the coefficient k_fc_. Capillary membranes are designed to be leaky, whereas alveolar membranes are designed to prevent leaks and they dominate the coupled system.

In Equation 3 when J_Vc_>0, the flow is edema, but when J_Vc_<0 it is clearance. Clinically, an important result is to set J_VA_ =J_Vc_=0 and solve for the critical blood pressure, p_c_=p_crit_, that achieves zero flow. The result is

(4) $$p_{crit}=0.735\;p_{AG}-0.15\;\sigma +0.8\left(\pi_{c}-\pi_{AL}\right)$$

where we substituted σ_c_=σ_A_=0.8, so π_i_ cancels out, and R = 0.01cm. When p_c_=p_v_>p_crit_, the central region flow is entirely edema: the larger the difference, the faster the flow. When p_c_=p_a_<p_crit_ it is entirely clearance: the larger the difference, the faster the flow. For p_c_ values in between p_a_ and p_v_, there can be bidirectional flow, i.e. upstream edema and downstream clearance. Equation 4 can be viewed as the alveolar edema equation.

## Discussion

A clinical goal is to lower p_c_ and/or increase p_crit_ to prevent edema and optimize clearance. Normal parameter values yield p_crit_=19.4mmHg. This compares well with clinical definitions of CPE indicated by PCWP>18-20mmHg, another model validation. In **Figure 1** (middle), CPE from setting p_a_=25mmHg and p_v_=22mmHg led to edema, because p_c_ exceeds normal p_crit_. However, increasing p_AG_ to 15cmH_2_O raises p_crit_ to 30.4mmHg, well above p_c_, causing clearance. In **Figure 2** (top), ARDS parameter values yield p_crit_=6 mmHg, explaining edema with normal p_c_. Then, increasing p_AG_ to 15cmH_2_O increased p_crit_ to 17mmHg, leading to clearance (**Figure 2**, middle). If only π_c_ is increased in ARDS, from 25 to 30mmHg, the new p_crit_=10mmHg yielding clearance **Figure 2** (bottom) but a small margin above p_c_. To boost p_crit_ in that situation, consider a combination ARDS therapy: set π_c_=30mmHg and add p_AG_=10cmH_2_O. That yields p_crit_=17.4mmHg and a higher clearance flow rate.

Three terms in Equation 4 (p_AG_, σ, and π_c_) are clinically adjustable parameters, which can be varied independently or in combinations as demonstrated above. They can be readily manipulated by the clinician at the bedside to improve clearance of the pulmonary interstitium. For example, p_c_ in both cardiogenic and non-cardiogenic pulmonary edema may be reduced with antihypertensive therapy whereas π_c_ may be increased with diuretics, intravenous albumin, hypertonic saline, or combinations (Khan et al., 2007; Uhlig et al., 2014; Li et al., 2023; Wang et al., 2023). Certainly, in the neonatal population, with varying results in the adult population (Filoche et al., 2015; Grotberg et al., 2017), surfactant replacement therapy can decrease surface tension, σ. Notably, the application of PEEP to increase p_AG_ can be individualized in both disease states to improve the direction of fluid-flow from alveolus to capillary. Indeed, the concept of prophylactic PEEP or CPAP is achievable with Equation 4. While all of these interventions have been studied individually, Equation 4 provides a basis for planning their relative impact.

In addition, the model finds that clearance flow rates from increased p_AG_ are much higher in ARDS compared with CPE for two reasons. First, increased k_fc_ and k_fA_ allow larger flows in either direction, particularly k_fA_ as seen in Equation 3. Second, the overall driving pressure for clearance is p_AG_-p_c_, which is greater in ARDS than CPE since p_c_ is normal in ARDS compared with CPE where it is elevated. Consequently, early aggressive PEEP/CPAP therapy in ARDS, or potential ARDS, could be prioritized before glucocorticoid therapy normalizes k_fc_ and k_fA_. In the wake of the COVID-19 pandemic, there have been numerous publications devoted to the personalization of PEEP in ARDS to optimize alveolar recruitment and limit alveolar overdistension using bedside tools such as esophageal manometry (P_es_) and electrical impedance tomography (EIT) (Jimenez et al., 2022; Grotberg et al., 2023). The application and titration of PEEP is often thought of as a method for alveolar recruitment to improve tidal volume distribution, lung mechanics, and pulmonary gas exchange. In “non-recruitable” patients, PEEP may over-distend open alveoli resulting in volutrauma and ventilator-induced lung injury. However, PEEP is not currently perceived as a method to improve clearance of alveolar and interstitial edema. The above model would allow the bedside clinician or first responder to calculate the p_AG_ required to raise p_crit_ and lower p_c_ to improve interstitial edema clearance or prevent edema formation. This could play an integral role in PEEP personalization in both cardiogenic and non-cardiogenic pulmonary edema alike.

## Data Availability

Publicly available datasets were analyzed in this study. This data can be found here: James B. Grotberg, grotberg@umich.edu.
